# Commensal Microbe-specific Activation of B2 Cell Subsets Contributes to Atherosclerosis Development Independently of Lipid Metabolism

**DOI:** 10.1016/j.ebiom.2016.10.030

**Published:** 2016-10-20

**Authors:** Lin Chen, Tomoaki Ishigami, Rie Nakashima-Sasaki, Tabito Kino, Hiroshi Doi, Shintaro Minegishi, Satoshi Umemura

**Affiliations:** Department of Medical Science and Cardio-Renal Medicine, Yokohama City University, Graduate School of Medicine, 3-9 Fukuura, Kanazawa-ku, Yokohama, Kanagawa, Japan

**Keywords:** Atherosclerosis, Inflammation, Intestinal microbiota, B2 cell, TLR signaling

## Abstract

The relation between B2 cells and commensal microbes during atherosclerosis remains largely unexplored. Here we show that under hyperlipidemic conditions intestinal microbiota resulted in recruitment and ectopic activation of B2 cells in perivascular adipose tissue, followed by an increase in circulating IgG, promoting disease development. In contrast, disruption of the intestinal microbiota by a broad-spectrum antibiotic cocktail (AVNM) led to the attenuation of atherosclerosis by suppressing B2 cells, despite the persistence of serum lipid abnormalities. Furthermore, pharmacological depletion of B2 cells with an anti-B2-cell surface CD23 antibody also attenuated commensal microbe-induced atherosclerosis. Moreover, expression analysis of TLR-signaling-related genes in the activated B2 cell subsets, assessed using the Toll-Like Receptor Signaling Pathway RT2 Profiler PCR Array, confirmed activation of the B2-cell autoantibody-production axis, which was associated with an increased capacity of B2 cells to bind to intestinal microbiota. Together, our findings reveal the critical role of commensal microbe-specific activation of B2 cells in the development of atherogenesis through lipid metabolism-independent mechanisms.

## Highlights

• Commensal microbe-specific activation of B2 cell plays a pivotal role in atherosclerosis through lipid metabolism-independent mechanism.
• B2-cell TLR signaling mediates microbiota-driven atherosclerosis.
• An anti-B2-cell antibody (CD23) was effective for preventing commensal microbe-derived atherosclerosis.

Atherosclerosis is among the leading causes of mortality and morbidity worldwide. Commensal microbe-derived atherosclerosis developing via metabolism-dependent mechanisms has received increasing attention. However, whether the effect of the intestinal microbiota on atherosclerosis is mediated by immune mechanisms remains unknown. The results of our study provide evidence for a pathway of immune activation in commensal microbe-derived atherosclerosis, which is driven by B2 cells that sense microbiota, and suggest that B2 cells may serve as a target for the development of therapeutic interventions.

## Graphical Abstract

Under hyperlipidemic condition, signals driven by the intestinal microbiota via TLRs signaling pathway cause B2 cells in spleen to become functionally active and activated B cells then modify responses such as antigen presentation (upregulation of costimulatory molecules-MHC class II molecules) and antibody production (generation of active antibodies IgG and IgG3), thereby contributing to the development of atherosclerosis. On the other hand, intestinal microbiota also resulted in recruitment and ectopic activation of B2 cells via TLRs signaling pathway in perivascular adipose tissue (PVAT) at the same time, and, subsequently, an increase in circulating IgG, led to the enhanced disease development.

**Figure:**
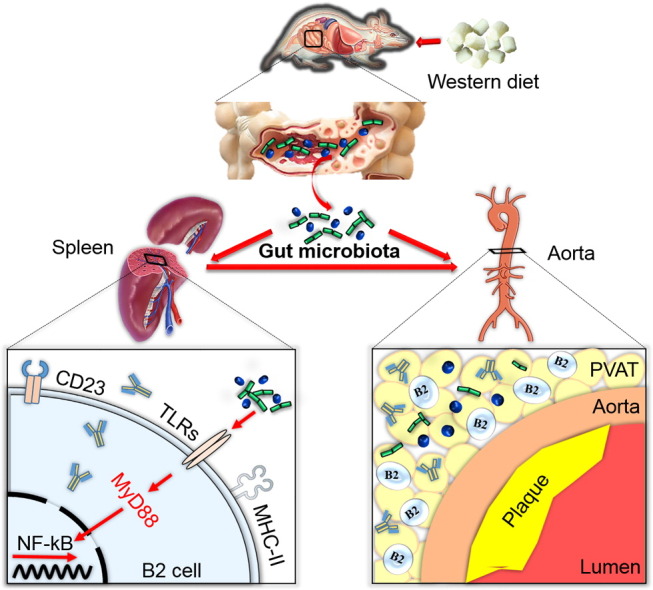

## 1 Introduction

Atherosclerosis is a major cause of harm to human health and a leading pathological contributor to cardiovascular morbidity and mortality worldwide. Studies during the past decade have highlighted the important role of the immune system in atherosclerosis (Libby, 2002, Wick et al., 2004). Consequently, reducing the associated inflammation that sustains the immune response has become an important target for scientific and future therapeutic investigations because lipid control alone does not effectively prevent the progression of atherosclerosis in some patients (Hilgendorf et al., 2014). However, despite a long-known association with atherosclerosis, B cells and immunoglobulins, important components of the immune system, have received relatively little attention. In particular, the roles of B2 cells, which represent the vast majority of B cells, including follicular (FO) as well as marginal zone (MZ) B cells, remained largely unexplored (Doran et al., 2012, Kyaw et al., 2010, Lipinski et al., 2011).

In general, FO B cells, which predominantly participate in T-cell-dependent (TD) antibody responses, might be endowed with proatherogenic potential (Nicoletti et al., 1998). In contrast to TD FO B cells, MZ B cells, situated peripherally to FO B cells, reside in the spleen (Victora, 2014), which serves as the interface with the circulation, and are located at the first line of defense against antigens (Martin and Kearney, 2002). MZ B cells predominantly give rise to rapid T-cell-independent (TI) antibody responses to antigens, producing TI antibodies (Puga et al., 2012). However, whether MZ B cells have atheroprotective properties similar to TI B cells (e.g., B1 cells) remains unknown (Ait-Oufella et al., 2010, Kyaw et al., 2011).

The spleen, the largest immune organ in the periphery, is the major B2 B-cell reservoir, which harbors about 80% of FO B cells and 10% of MZ B cells among all splenic B cells (Tsiantoulas et al., 2014). However, it should be noted that spleen-associated immune activity protects against atherosclerosis (Emtiazy et al., 2013, Lammers et al., 2012, Rezende et al., 2011), and removal of the spleen, which results in accelerated atherosclerosis, has been shown to deplete B1a cells from the peritoneum, followed by a strong decrease in plasma immunoglobulin (Ig) M titers (Kyaw et al., 2011, Wardemann et al., 2002). Moreover, it should be emphasized that FO B cells and MZ B cells are also missing after splenectomy. In addition, at present, there is contradictory evidence concerning the role of B2 cells in atherogenesis after adoptive transfer of splenic B2 cells (Doran et al., 2012, Kyaw et al., 2010, Lipinski et al., 2011), and dissection of the functions of individual B2-cell subsets (MZ versus FO) and separation of the intrinsic biological properties of these B2 cells from effects mediated by the antibodies they secrete during atherogenesis have yet to be performed (Tsiantoulas et al., 2014).

On the other hand, as one important source of antigens, recent studies have suggested that commensal microbes might exert a substantial impact on atherosclerosis (Brown and Hazen, 2015, Koren et al., 2011, Serino et al., 2014, Tang and Hazen, 2014). Remarkably, commensal microbes play an essential role in the activation of MZ B cells and B2 cell-mediated IgG antibody production (Balázs et al., 2002, Cerutti et al., 2012, Martin and Kearney, 2002, Puga et al., 2012, Vinuesa and Chang, 2013, Weill et al., 2009). However, whether these effects contribute to the development of atherosclerosis remains largely unexplored. In parallel with these findings, our previous clinical research revealed that many autoantibodies differing from those found in chronic autoimmune diseases are associated with atherosclerosis (Ishigami et al., 2013). Meanwhile, previous studies have reported that human antibodies cross-react with outer membrane proteins of bacteria as well as with proteins involved in atherogenesis (Canducci et al., 2012). However, the link between B2 cells and commensal microbes in the development of atherosclerosis has not yet been reported.

To address the above issues, we therefore set out to characterize the B2-cell subsets and investigated the role of commensal gut flora in triggering B2-cell response during atherogenesis. B2 cells were shown to play an important immunological role in atherosclerosis and may serve as a target for the development of therapeutic interventions.

## 2 Methods

### 2.1 Mice

ApoE KO mice (on the C57BL/6J background) were a generous gift from Dr. Hashimoto (Yokohama City University Graduate School of Medicine, Yokohama, Japan). C57BL/6J mice were purchased from the Oriental Yeast (Tokyo, Japan). The number of mice per group used in each experiment is annotated in the corresponding figure legend as n. All mice admitted to the study survived the intended duration of the study, and only 5-week-old male mice were used for experiments. Until required for experiments, the mice were provided with a standard diet and tap water ad libitum. All of the animal studies were conducted in accordance with the animal care guidelines of Yokohama City University Graduate School of Medicine.

### 2.2 Commensal Bacteria Depletion

The role of the microflora was investigated by treating mice with antibiotics. For experiments, 5-week-old male ApoE KO mice were assigned to the control group or the antibiotic treatment (AT) group. Mice in the AT group were treated with a broad-spectrum antibiotic cocktail AVNM, which contained 0.5 g/L vancomycin, 1 g/L neomycin sulfate, 1 g/L metronidazole and 1 g/L ampicillin. The antibiotic cocktail was provided in drinking water for 1 week before beginning a Western-type diet (WD) and continued during the experimental period (Croswell et al., 2009, Rakoff-Nahoum et al., 2004, Wang et al., 2011). Antibiotics were purchased from Sigma or Wako (Tokyo, Japan). Drinking water containing antibiotics was changed every 3–4 days.

### 2.3 Active Induction of Atherosclerosis

Mice were provided with a WD (containing 21.22% [g/100 g] fat, 17.01% protein, 48.48% carbohydrate, and 0.15% cholesterol; Oriental Yeast, Tokyo, Japan) and a normal chow diet (ND) respectively after 1 week of AT and were maintained on the diet for 8 weeks.

### 2.4 Micro-computed Tomography (CT)

Micro-CT scanning was performed to evaluate visceral and subcutaneous fat before sacrifice. Anesthetized mice were imaged on the in vivo micro X-ray CT system R_mCT2 (Rigaku Co., Tokyo, Japan). The scan conditions used were as follows: voltage, 90 kV; current, 160 μA; FOV, 60 mm; scan technique, 17 s. Whole visceral and subcutaneous fat volumes were traced. Quantitative measurements were performed using Metabolic Analysis Software (Rigaku Co., Tokyo, Japan).

### 2.5 Quantification of Atherosclerosis and Histologic Analysis

After 8 weeks of consuming a WD, hearts and aortas were removed, and atherosclerotic lesions were quantified by cross-sectional analysis of the aortic root and by en face analysis of the whole aorta. Hearts were embedded in paraffin, and serial 5 μm sections were cut from the beginning of the 3 aortic leaflets to the ascending aorta and then stained with hematoxylin and eosin (H&E) stain. Afterwards, the aortas were fixed in 10% formalin, opened longitudinally, and stained for lipid depositions with Oil Red O solution (Sigma-Aldrich, USA). Lesion areas were calculated using the Image J program (National Institutes of Health, USA; http://rsb.info.nih.gov/ij). For plaque measurements, briefly, sections that captured the maximum lesion area were used to compare lesion sizes between study groups. Plaque areas and the total vessel area were determined, and the relative plaque extension was expressed as a percentage of the total vessel area.

### 2.6 Histology and Immunohistochemistry

Freshly isolated spleens were weighed, imaged and then formalin-fixed, paraffin-embedded, and sectioned (5 μm). Spleen architecture was evaluated after H&E staining. Numbers of germinal centers were assessed using Image J software. Tissue samples from perivascular adipose tissue (PVAT) were processed and embedded in paraffin according to standard procedures. Sections of 5 μm were stained with H&E stain or CD23 antibody (Product code: ab185807, Abcam, USA). Fat cell area on H&E-stained slides was measured using Image J software, quantifying two different tissue sections per mouse (at least 3 mice with at least 100 fat cells in each image). In addition, CD23 antibody specific for B2 cells was used to stain B2 cells in PVAT. The percentage area of CD23 positive field was assessed in a blinded manner using Image J software.

### 2.7 Preparation of Tissues for Flow Cytometry and Cell Sorting

Splenocytes and PVAT cells were harvested and processed for flow cytometry. In brief, cells were released from spleen by nicking the capsule and gently rotating two microscope slides. Remaining erythrocytes were removed by incubation with a red blood cell lysing buffer (Product No. R7757, Sigma). Meanwhile, complete PVAT cells were freed from aortas at 4 × magnification and digested by collagenase type 4 (0.5 mg/mL) (Catalog No. LS004186, Worthington Biochemicals) for 30 min at 37 °C; total PVAT cells were then collected. Single-cell suspensions were filtered through a nylon mesh (100 μm, BD Biosciences) and stained either with a mixture of fluorochrome-conjugated anti-mouse monoclonal antibodies CD21 (APC, Catalog No. 561770, BD Biosciences), CD23 (PE, Catalog No. 561773, BD Biosciences), CD45R/B220 (FITC, Catalog No. 561877, BD Biosciences) or CD45 (FITC, Catalog No. 561088, BD Biosciences), F4/80 (PE, Catalog No. 565410, BD Biosciences), CD3e (APC, Catalog No. 557306, BD Biosciences) for 30 min on ice. Then, the cells were resuspended in a fixable 49, 6-diamino-2-phenylindole (DAPI, Catalog No. P4864, Sigma) live/dead stain diluted with flow cytometry staining buffer (Catalog No. 00-4222-57, eBioscience). Flow cytometric analyses were performed on a MoFlo Astrios (Beckman Coulter), and the results analyzed by software ver. 6.2.3. Dead cells were excluded by FSC, SSC, and DAPI staining. Distinct B-cell populations, T cells, and macrophages were identified upon cell surface expression of the indicated antigens. Specifically, FO B cells were identified as B220^+^ CD21^+/lo^CD23^+^ cells, MZ B cells were identified as B220^+^ CD21^high^CD23^−/lo^ cells, transitional B cells were identified as B220^+^ CD21^−^ CD23^−^ cells, and B1 cells were identified as B220^low^CD23^−^ cells. In addition, T cells were identified as CD45^+^ CD3e^+^ F4/80^−^ cells, and macrophages were identified as CD45^+^ F4/80^+^ CD3e^−^ cells. For cell sorting experiments, FO B and MZ B cells were sorted to higher than 99% purity from their parent gate.

### 2.8 Real-time Polymerase-chain-reaction (RT-PCR) Analysis

Total RNA was isolated from the pooled sorted cells using RiboPure Kit (Catalog No. AM1924, Life Technologies) or RNeasy Plus Micro Kit (Catalog No. 74034, QIAGEN) according to the manufacturer's protocol. 1 μg of RNA was reverse-transcribed to cDNA using a High Capacity RNA-to-cDNA Kit (Catalog No. 4387406, Applied Biosystems) according to the manufacturer's protocol. The expression levels of major histocompatibility complex (MHC)-class II were analyzed by RT-PCR using SYBR-Green (Catalog No. 4309155, Applied Biosystems) on an ABI 7500 RT-PCR machine according to the manufacturers' recommendations. The relative mRNA expression levels were determined using glyceraldehyde-3-phosphate dehydrogenase (GAPDH) as housekeeping gene and the 2[− ΔΔC (T)] method. The following primers were used: mouse MHC-class II (LeibundGut-Landmann et al., 2004): I-Aa forward, 5′-AAATTCCACCCCAGCTACCAAT-3′; I-Aa reverse, 5′-GCTGACCCAGCAGCACAG-3′. In addition, to analyze toll-like receptor (TLR) signaling genes, mouse TLR signaling pathway RT2 Profiler PCR arrays (Catalog No. PAMM-018Z, Qiagen) were used to profile the expression of 84 genes related to TLR signaling. Total RNA from pooled sorted cells was used, and single-stranded cDNA was synthesized from 25 ng of total RNA by using an RT2 PreAMP cDNA synthesis kit and RT2 PreAMP Pathway Primer Mix (Catalog No. 330451; PBM-018Z, Qiagen). The cDNAs were mixed with RT2 SYBR Green ROX qPCR Mastermix, and RT-PCR performed in accordance with the manufacturer's instructions. Thermal cycling and fluorescence detection were performed using an ABI 7500 Sequence Detection System (Applied Biosystems). Data were quantified using RT2 Profiler PCR array data analysis software, version 3.5 (Qiagen, http://pcrdataanalysis.Sabiosciences.Com/pcr/arrayanalysis.php?target=upload). Only Ct values < 35 were included in the calculations, and gene expression was related to the mean expression of β2 microglobulin (β2M) and GAPDH as housekeeping genes, since these were the two most stable of the five housekeeping genes included in the array, and the transcripts of interest were reported as log2 fold changes and average expression, and the expressions of TLRs regulated transcripts were compared between the groups as heat maps.

### 2.9 Quantification of Plasma Parameters

Whole blood was harvested from mice at the time of sacrifice by right ventricular puncture. Total cholesterol, low-density lipoprotein cholesterol (LDL-C), high-density lipoprotein cholesterol (HDL-C) and triglyceride were determined by SRL Inc. (SRL Inc., Tokyo, Japan) (Hashimoto et al., 2006, Hosoi et al., 2010, Irie et al., 2015) with LDL-cholesterol Kit (Catalog No. 30173000, Sekisui Medical CO., Tokyo, Japan), HDL-cholesterol Kit (Catalog No. 30169000, Sekisui Medical CO., Tokyo, Japan), Cholesterol Kit (Catalog No. 460-44301, Wako, Tokyo, Japan) and Triglyceride Kit (Catalog No. 464-44201, Wako, Tokyo, Japan). LDL-cholesterol Kit and HDL-cholesterol Kit provided the credible assay methods of LDL-C and HDL-C, and have been certified through the certification program of the Centers for Disease Control and Prevention (CDC) and Cholesterol Reference Method Laboratory Network (CRMLN). (http://www.cdc.gov/labstandards/crmln_clinical.html. and http://www.sekisuimedical.jp/business/diagnostics/biochemistry/ldl/attention.html). Immunoglobulin measurement: Total serum IgG and IgG3 were measured in diluted serum with the Mouse IgG ELISA Kit (Bethyl Laboratories, E99-131) and Mouse IgG3 ELISA Kit (Bethyl Laboratories, E99-111) according to the manufacturer's instructions. Data were expressed as microgram per milliliter, based on the standard curves of isotype standards.

### 2.10 Depletion of B2 Cells by Antibody Treatment

To determine the possible involvement of commensal microbe-specific activation of B2 cell subsets in atherogenesis development, we administered a neutralizing anti-CD23 antibody (Purified Rat Anti-Mouse CD23, BD Pharmingen™, B3B4) to ablate B2 cells or saline (to serve as a control) 50 μg i.p. (0.5 μg/μL; 100 μL) 1 week prior to starting WD treatment and then 50 μg i.p. each week during the induction of atherosclerosis (Coyle et al., 1996, Dasic et al., 1999).

### 2.11 Statistical Analysis

Statistical analyses were performed with the SPSS 22 software programs. Differences between groups were considered to be statistically significant when *p* < 0.05 using the appropriate tests. We used one-way ANOVA tests followed by Tukey post hoc tests when values were normally distributed or nonparametric Kruskal–Wallis tests when values were not normally distributed, as specified in the figure legends.

## 3 Results

### 3.1 Gut Commensal Microflora Promote the Development of Atherosclerosis

**Fig. 1:**
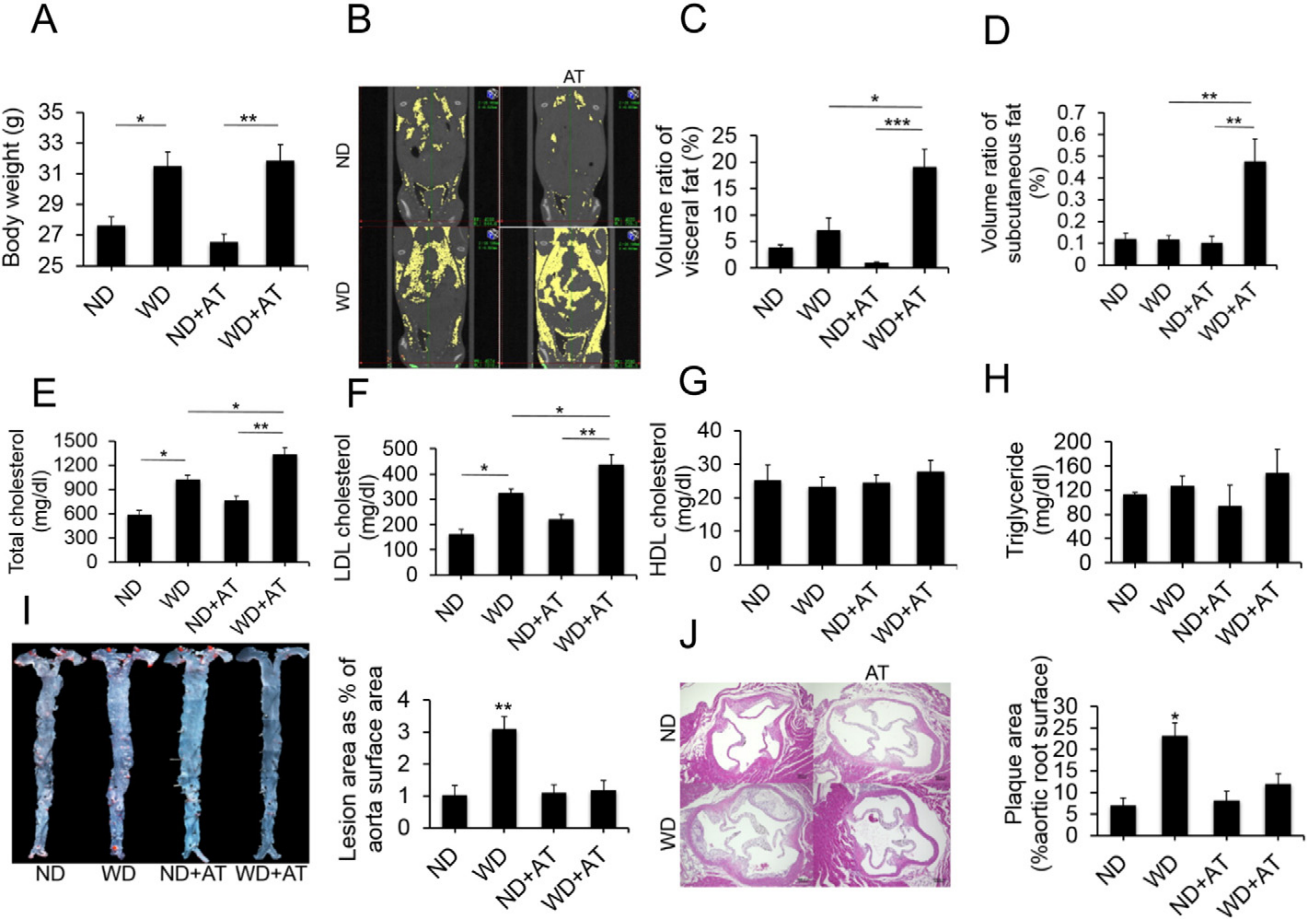
Antibiotic treatment mice are resistant to diet-induced atherosclerosis. Mice given a Western diet or normal chow diet with or without antibiotic treatment for 8 weeks. (A) Body weights. AT: antibiotic treatment; WD: Western diet; ND: normal chow diet. Data pooled from three independent experiments (means ± SEM. n = 7 for each group). (B–D) Representative micro-CT images of fat (B) and quantification of total percent of visceral fat volume (C) and subcutaneous fat volume (D) are shown. Data are representative of three independent experiments (means ± SEM. n = 7 for each group) *p < 0.05, **p < 0.01, ***p < 0.001 according to the post hoc ANOVA statistical analysis. (E–H) Serum levels of total cholesterol (E), LDL (F) and HDL cholesterol (G), and triglyceride (H) were assessed. LDL: low-density lipoprotein; HDL: high-density lipoprotein. Results are presented as means ± SEM. n = 7 per group, *p < 0.05, **p < 0.001. (I and J) Aortic lesion size was assessed by oil red O (I) and HE staining (J). Representative images and quantification of oil red O staining of aortas prepared en face are shown in I (% of aortic surface area, n = 12 for each group). Representative images and quantification of maximum lesion area within the aortic root sections as determined by HE staining are shown in J (n = 12 for each group). Scale bars represent 100 μm. Data pooled from three independent experiments. *p < 0.05, **p = 0.001, comparing WD vs. the other groups by ANOVA.

To assess the impact of gut commensal microflora on atherosclerosis, AT was used to eliminate the gut commensal microflora. Although we did not perform bacterial culture of feces or RT-PCR amplification of 16S rRNA gene sequences to quantitatively analyze the total biota, the results of previous studies indicate that a broad-spectrum antibiotic cocktail (AVNM) has sufficient activity to deplete all detectable commensal bacteria (Rakoff-Nahoum et al., 2004, Wang et al., 2011), and macroscopic examination of the GI tract revealed characteristic secondary findings associated with the depletion of gut commensal bacteria, such as marked swelling of the cecum ascribed to the accumulation of undigested mucus (Barthel et al., 2003, Croswell et al., 2009). These findings indicated a reduction in the levels of colonizing bacteria (not shown). The body weights in the WD group and ND group with or without AT were monitored weekly, and the results are shown in Fig. 1A. Body weight differed significantly between the WD group and ND group (*p* < 0.05). However, there was no obvious difference in body weight between the WD-fed mice and the age-matched WD-AT mice. This finding indicated that elimination of the intestinal microbiota by AT had no impact on WD-induced weight gain. Intriguingly, AT resulted in increased visceral and subcutaneous fat during WD feeding, whereas micro-CT analysis at the end of the 8-week diet study showed no changes in visceral or subcutaneous fat in ApoE KO mice given a WD as compared with those given a ND. However, after AT, significant increases in visceral fat (*p* < 0.05) and subcutaneous fat (*p* < 0.01) were seen only in mice given a WD (Fig. 1B–D). The increased visceral and subcutaneous fat gain observed in WD-AT mice prompted us to test whether this phenomenon was accompanied by metabolic alterations. Indeed, we detected dyslipidemia in the WD-AT mice. In parallel with the CT results, the serum lipid levels increased significantly in the WD-AT mice, and the serum total cholesterol and LDL-C levels were significantly higher in the WD-AT mice than in the WD-fed mice (*p* < 0.05) (Fig. 1E–H). Although the changes in the plasma cholesterol levels were obvious, atherosclerotic lesion size, which was determined by oil red O staining of whole aortas after 8 weeks of a WD, surprisingly indicated that AT significantly decreased en face atherosclerotic lesion size associated with a WD (*p* < 0.001) (Fig. 1I). Histological examination of the aortic roots confirmed a similar decrease in atherosclerotic lesion size (*p* < 0.05) (Fig. 1J), as well as in and macrophage and T cell-rich areas (data not shown). To assess whether these phenotypes were specific to ApoE KO mice, the same analyses were performed in normal C57BL/6J mice, and as expected similar results were obtained (Supplementary Fig. S1). Analysis of the relation between plaque size and total cholesterol levels showed no correlation between these variables. This finding indicates that proatherogenic effects are related to gut commensal microflora and do not solely depend on hypercholesterolemia. Collectively, these data suggest that gut commensal microflora also can promote atherosclerosis development through lipid metabolism-independent mechanism.

### 3.2 AT Reduced Atherosclerosis by Abolishing the WD-induced Increase in FO B Cells

Beside the spleen, B cells are also known to reside in the adventitial layer of vessels (Gräbner et al., 2009, Hamze et al., 2013). On the other hand, the role of gut microbes in regulating the immune system through metabolism-independent pathways has been identified (Brown and Hazen, 2015, Drosos et al., 2015). Therefore, to test the hypothesis that the impact of intestinal microbiota on atherosclerosis is via activation of B2 cells, we initially analyzed B2-cell populations in spleen and PVAT.

**Fig. 2:**
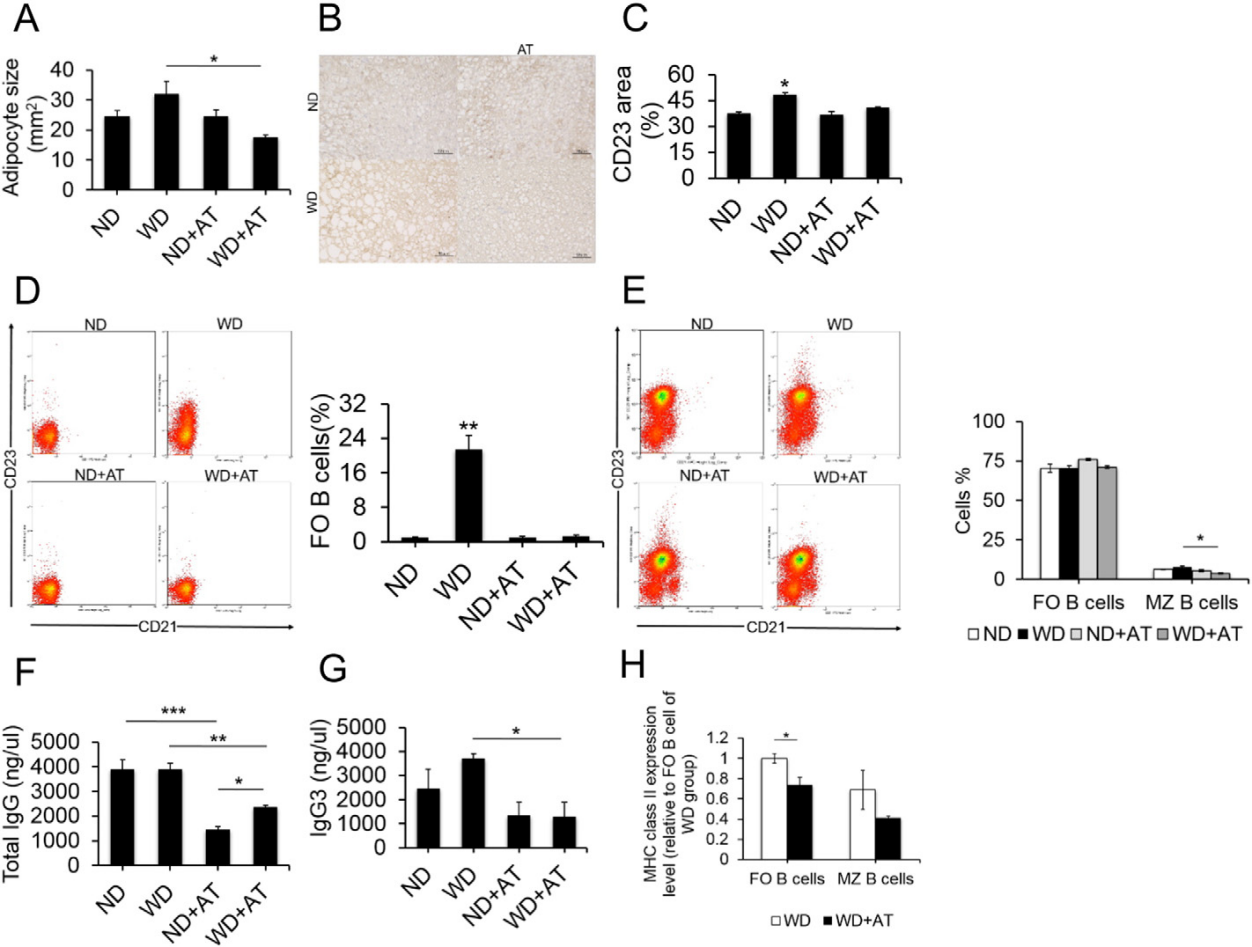
Antibiotic treatment controls atherosclerosis by blocking B2 cells expansion. At the end of 8 weeks of a Western diet (WD) or normal chow diet (ND), the effects of antibiotic treatment (AT) on the typical features of PVAT expansion and accumulation of CD23 positive cells are shown in A–C. (A) Mean adipocyte diameter was quantified in PVAT sections. The surface area of at least 250 adipocytes per image was counted, and a total of seven images per PVAT were analyzed with ImageJ software. Data represent means ± SEM from n = 7 experiments. *p < 0.001 for 1-way ANOVA with Tukey corrections. (B and C) Representative images (B) and quantification of CD23-positive staining in PVAT sections (C). Scale bars represent 50 μm. Values are presented as means ± SEM. *p < 0.001, comparing WD vs. the other groups by ANOVA. (D and E) Flow cytometry analysis of (D) follicular (FO) B cells of PVAT, (E) FO B cells and marginal zone (MZ) B cells in spleen, respectively (n = 6 mice per group). Data from 14 mice pooled from 3 independent experiments. Cell counts are presented as means ± SEM. *p < 0.01, **p < 0.001. (F and G) Concentrations of total IgG (F), IgG3 (G) were assessed by ELISA. Values are presented as means ± SEM. n = 6 per group from 3 independent experiments, *p < 0.05, **p < 0.01, ***p < 0.001 by Tukey post-hoc test. (H) RNA expression of MHC class II analyzed in purified, cell-sorted B220^+^ CD21^+/lo^CD23^+^ (FO B cells), B220^+^ CD21^high^CD23^−/lo^ (MZ B cells) in spleen digest from WD and WD-AT by real-time polymerase chain reaction and normalized to GAPDH. Results are presented as means ± SEM. fold change of 2^△Ct^, *p < 0.05, n = 7 per group.

In healthy individuals, adipose tissue expansion occurs by enlargement of the fat pad mass through enhanced recruitment of adipocyte precursor cells that differentiate into small adipocytes. In contrast, pathological expansion of adipose tissue is characterized by rapid growth of the fat pad through enlargement of existing fat cells. Such pathological expansion is associated with chronic inflammation (Sun et al., 2011). Therefore, the observed metabolic syndrome in WD-fed and WD-AT mice indicated that pathological adipose tissue expansion might occur in these mice. As expected, adipocytes in the PVAT of WD-fed mice were larger, less organized, and more loosely packed as compared with adipocytes in the control group (*p* < 0.001; Fig. 2A–B). However, as shown in Fig. 2A–B, typical features of adipocytes in the PVAT of WD-AT mice did not significantly differ from those in ND-fed mice. Moreover, we examined the effect of the elimination of gut commensal microflora on the accumulation of B2 cells in the PVAT of WD-fed mice. Consistent with the above finding, WD-fed mice showed a significant increase in the accumulation of CD23-positive cells as assessed by immunohistochemical analysis (*p* < 0.001; Fig. 2B–C). Conversely, the numbers of CD23-positive cells were equivalent in the WD-AT and control group (Fig. 2B–C), suggesting that pronounced B2 cell infiltration was augmented in the PVAT of WD-fed mice and completely abolished along with the depletion of all detectable commensal bacteria. These findings support the notion that accumulating B2 cells are essential contributors to the induction of atherosclerosis by intestinal microbiota.

To further confirm the above findings, we studied the B2 cells of PVAT by flow cytometry. Representative flow cytometry plots along with our gating strategy to differentiate these subsets are depicted in Fig. 2D. In the PVAT, the proportion of activated FO B cells dramatically increased in the mice given a WD as compared with those given a ND (*p* < 0.001). However, after microbiota depletion by AT, the WD-induced increase in FO B cells was abolished (*p* < 0.001) which was consistent with the above finding. Concomitantly, we also investigated B2 cells in the spleen, the major B cell reservoir. Although there was no significant difference in spleen weight (Supplementary Fig. S2A), and WD-feeding and AT did not modify the overall percentage of FO B cells in spleen (Fig. 2E) and was not associated with changes in splenic architecture indicative of FO B cell activation on histological staining (Supplementary Fig. S2B–C), the proportion of MZ B cells was significantly decreased in the mice given a WD after AT (*p* < 0.01; Fig. 2E). Furthermore, we also confirmed the above results in normal C57BL/6J mice (Supplementary Fig. S3). Remarkably, the numbers of activated FO B cells were expanded in the PVAT of WD-fed mice, whereas AVNM-mediated microbiota depletion led to a significant reduction in FO B2 cells, and the expansion was completely abrogated in C57BL/6J mice (Supplementary Fig. S3). In addition, as the primary function of activated B cells is to produce antibodies, we also investigated whether AT altered circulating IgG titers (Fig. 2F–G). Although the increased number of FO B cells was not associated with increased total IgG titers in the serum of the mice given a WD as compared with the mice given a ND, after AT, the total IgG titers in the serum sharply decreased, followed by reductions in FO B cells and MZ B cells (*p* < 0.01; Fig. 2F). Moreover, analyses of IgG3, which is a specific subclass of IgG and mainly produced by MZ B cells via a TI pathway under preimmune conditions or after immunization (Guinamard et al., 2000, Panda et al., 2013), revealed that WD-AT mice displayed decreased serum levels of IgG3 as compared with WD-fed mice (*p* < 0.05; Fig. 2G). Moreover, there was also a trend toward increased serum levels of IgG3 in WD-fed mice relative to the ND-fed mice, although the difference did not reach statistical significance. We further examined the mRNA expression levels of MHC-class II in B2 cells, which can mediate antigen presentation and represent another important function of activated B cells. Although there was no difference in spleen FO B2 cells between the WD group and WD-AT group on flow cytometric analysis (Fig. 2E), as shown in Fig. 2H, AT significantly decreased MHC-class II expression in spleen FO B2 cells of WD-fed mice. Therefore, these data suggest that intestinal microbiota-induced atherosclerosis is characterized by B2-cell activation.

### 3.3 Intestinal Microbiota Potentiates Activation of B2-cell TLR Signaling and Inflammatory Gene Expression during Atherogenesis

**Fig. 3:**
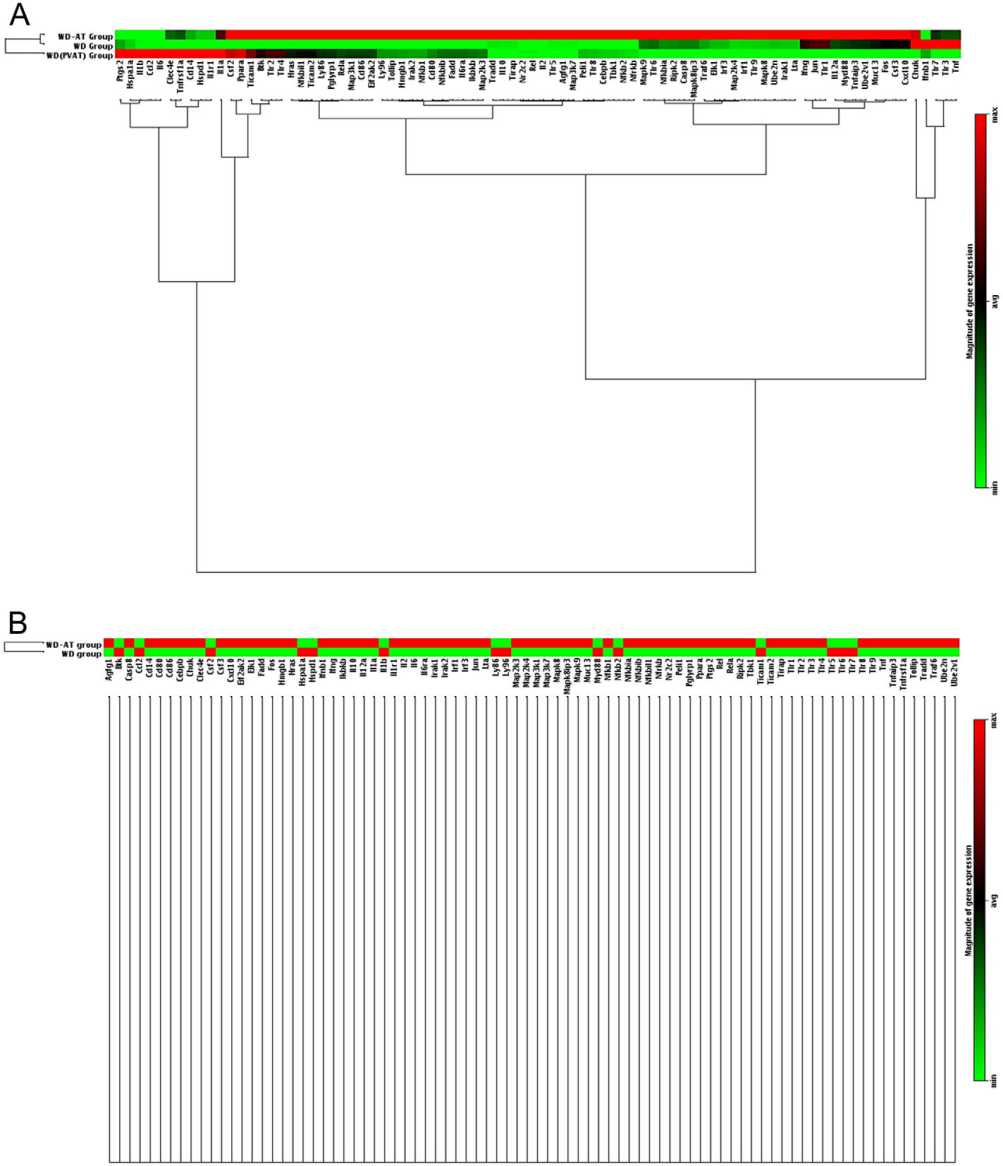
Distinct gene expression profiles associated with TLR signaling pathway in B2 cells following WD and AT. Messenger RNA preparations of sorted FO B cells from PVAT and spleen and MZ B cells from spleen were analyzed by mouse toll-like receptor signaling pathway RT2 Profiler PCR arrays. Gene expression reportedly associated with TLR signaling pathway was compared among the indicated FO B2 cell groups (A) and indicated MZ B2 cell groups (B), respectively. Results are displayed as heat maps. Red, max (magnitude of gene expression); green, min (magnitude of gene expression).

B cells exhibit variations in TLR expression patterns, which may directly transduce microbiota-derived signals. Alternatively, microbiota-derived signals via TLRs can modify B cell responses such as antibody production, antigen presentation, and cytokine secretion (Nickerson et al., 2010, Pasare and Medzhitov, 2005, Rawlings et al., 2012). The aforementioned results indicate that antibody responses are diminished via elimination of intestinal microbiota in WD-fed mice. Therefore, to further characterize these B2 cells and decipher whether B2-cell TLR signaling contributes to the microbiota-induced atherosclerosis observed in WD-fed mice, we isolated B2 cells from spleen and PVAT and quantified gene expression of TLR signaling. As expected, comparisons of the WD and WD-AT groups revealed a substantial different in gene expression profiles (Fig. 3A and Table 1, Table 2). Although TLR expression was unaffected (Supplementary Table S1), we found that the gene expression levels of proinflammatory cytokines were indeed elevated in FO B2 cells from PVAT of WD-fed mice. As the most striking difference, the transcription of CD14, which encodes the molecule surface antigen CD14, a molecule required for B-cell function, was significantly up-regulated in FO B cells of PVAT. Moreover, previous studies revealed that CD14 is a component of the innate immune system and acts as a co-receptor along with TLR 2 and TLR 4 for the detection of bacterial lipopolysaccharide (LPS) (Heumann et al., 2001, Tasaka et al., 2003). In addition, the genes encoding inflammation-activated related molecules, including chemokine ligand 2 (*Ccl2*), heat shock 70 kDa protein 1a (*Hspa1a*), interleukin 1 beta (*IL1β*), interleukin 1 receptor, type 1 (*IIl1r1*), prostaglandin-endoperoxide synthase 2 (*Ptgs2*), and tumor necrosis factor receptor superfamily member 1a (*Tnfrsf1a*), were also up-regulated. In contrast to FO B2 cells from the spleen of WD-fed mice, the expressions of CCAAT/enhancer binding protein, beta (*Cebpb*) and NF-kappa-B inhibitor beta (*Nfkbib*), which are negative regulators of TLR signaling, were induced in WD-AT mice, whereas the expressions of the inflammation-activated related genes *Hspa1a* and *Ptgs2* were dramatically reduced (Table 1). These findings strongly suggest that the observed changes resulted from the elimination of the intestinal microbiota, which promotes atherosclerosis via activation of B2-cell TLR signaling pathway. B2-cell TLR signaling thus mediates microbiota-driven atherosclerosis. Taken together, these findings support a specific role of TLR signaling in B2 cells during microbiota-driven atherosclerosis.

### 3.4 B2-cell Deficiency Attenuates Microbiota–induced Atherosclerosis

**Fig. 4:**
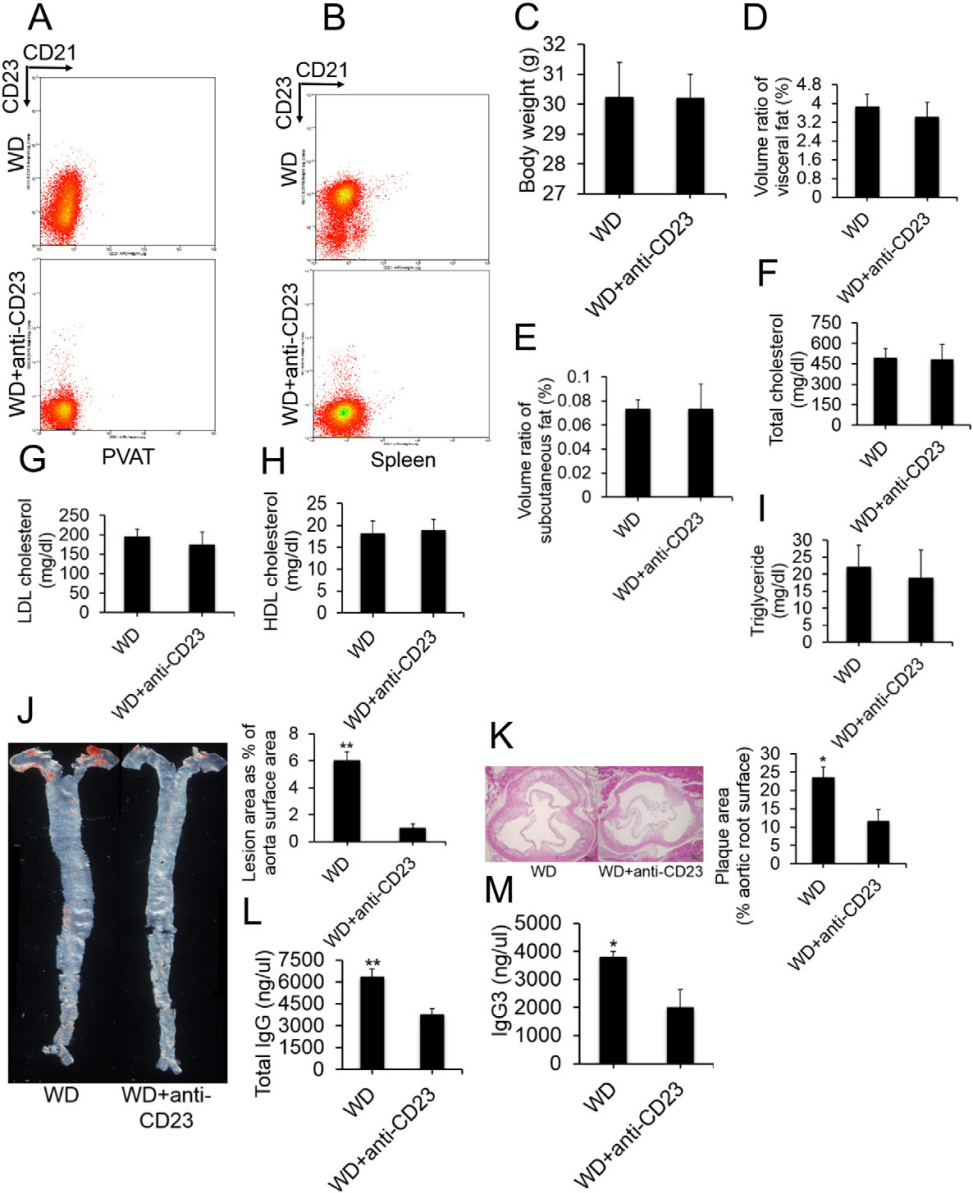
Pharmacological depletion of B2 cells protects mice from atherosclerosis. (A and B) Representative flow cytometric plots of B2 cell numbers in the PVAT (A) and spleens (B) of mice treated with a mouse specific CD23 antibody or saline (n = 6 per group). (C) Body weights measured at the end of 8 weeks of a Western diet in mice treated with a mouse specific CD23 antibody or saline. Values are presented as means ± SEM from n = 6 experiments. (D and E) Micro-CT of total percent of visceral fat volume (D) and subcutaneous fat volume (E). Data are representative as means ± SEM. n = 6 for each group. (F–I) Serum levels of total cholesterol (F), LDL (G) and HDL (H) and triglyceride (I) were assessed. LDL: low-density lipoprotein; HDL: high-density lipoprotein. Results are presented as means ± SEM. n = 6 per group. (J and K) Effect of an anti-mouse CD23 antibody on plaque formation is shown in J–K. (J) Representative images and quantification of oil red O staining of aortas prepared en face. (K) Representative HE staining of aortic root sections and quantification of maximum lesion area. Group data (means ± SEM) from n = 6 experiments. Scale bar, 100 μm. *p < 0.05, **p < 0.001 for 1-way ANOVA with Tukey's corrections. (L and M) Concentrations of total IgG (L) and IgG3 (M) in serum from mice treated with a mouse specific CD23 antibody or saline. Values are presented as means ± SEM from n = 6 experiments. *p < 0.05, **p < 0.01 for 1-way ANOVA with Tukey's corrections.

Because intestinal microbiota depletion may influence the development of atherosclerosis by reducing the number of activated B2 cells, we further investigated whether B2-cell deficiency might afford protection against microbiota-induced atherosclerosis. A cohort of WD-fed mice was pretreated with a B2-cell–depleting agent, anti-mouse CD23 antibody. Intraperitoneal injections of anti-CD23 antibody were started 1 week before the development of atherosclerosis. The control group for these experiments comprised mice pretreated with saline. As expected, mice that received the mouse-specific CD23 antibody had far fewer B2 cells in their spleens and PVAT than did mice treated with saline (Fig. 4A–B). There were no changes in other cell populations (Supplementary Fig. S4). Furthermore, we found that WD-fed mice treated with anti-CD23 antibody gained weight in association with increased visceral and subcutaneous fat and serum lipid levels, similar to the WD-fed controls (Fig. 4C–I). However, after 8 weeks of WD, we compared plaque in WD-fed mice versus WD-fed plus anti-CD23 antibody-treated mice. WD-fed plus anti-CD23 antibody-treated mice exhibited a marked reduction in plaque formation as compared with that in WD-fed mice (Fig. 4J–K). At the same time, serum IgG and IgG3 levels were found to be elevated only in WD-fed mice not treated with antibody (Fig. 4L–M). These results confirmed that potential triggering of atherosclerosis by microbiota requires initial help from B2 cells. Altogether, these data indicate that microbiota aggravates atherosclerosis by stimulating activated B2-cell production and shifting the host response toward TH1-associated immunity.

## 4 Discussion

Studies presented here provide evidence supporting a critical role of commensal microbe-specific activation of B2 cell subsets in the development of atherogenesis through lipid metabolism-independent mechanism. First, we showed that WD-dependent atherosclerosis in mice is associated with B2 cell activation in PVAT, along with increases in total IgG and IgG3 levels in serum. However, microbiota depletion with broad-spectrum antibiotics (AVNM) resulted in significant and selective reductions in the numbers of FO B2 cells in PVAT and MZ B2 cells in spleen. Furthermore, we found that activation of B2 cell TLR signaling-related genes was associated with an increased capacity of B2 cells to bind the intestinal microbiota. Finally, as proof of concept that B2 cells can be targeted therapeutically to reduce atherosclerosis, we demonstrated that an anti-B2-cell antibody (CD23) effectively prevented commensal microbe-derived atherosclerosis in response to hyperlipidemia.

The intriguing relationship between commensal microbes and atherosclerosis has received increasing attention over the past few years. However, the specific mechanisms whereby commensal microbes regulate the development of atherosclerosis are just beginning to be elucidated (Brown and Hazen, 2015, Koren et al., 2011, Serino et al., 2014, Tang and Hazen, 2014). Recently, Spence et al. found that a number of metabolites from proteins/amino acids in the diet, including p-cresyl sulfate, indoxyl sulfate, and others (Spence et al., 2016), might contribute to development of cardiovascular disease (CVD). Previous studies have identified the pathway linking dietary lipid intake, intestinal microflora, and atherosclerosis. These studies indicated that increasing the dietary intake of precursors of toxic metabolic products of the intestinal microbiome, such as the trimethylamine-*N*-oxide from phosphatidylcholine and other forms of choline and carnitine (Koeth et al., 2013, Tang et al., 2013, Wang et al., 2011), was associated with CVD. The pathway of the metabolism of these toxic metabolic products of the intestinal microbiome represents a unique additional nutritional contribution to the pathogenesis of CVD. In fact, numerous studies have shown that commensal microbe-derived metabolites can act as hormones modulating CVD risk (Brown and Hazen, 2015). Metabolism-independent pathways, in particular the role of the immune system in commensal microbe-derived atherosclerosis, remain largely unexplored.

Our study showed that serum lipid levels were significantly increased in WD-fed mice regardless of AT or not, which is in line with previous observations (Fu et al., 2015, Le Chatelier et al., 2013, Velagapudi et al., 2010). However, it remains unclear how the gut microbiota affects serum lipid metabolism and systemic lipid metabolism in adipose tissue. It is thought that hyperlipidemia (especially LDL-C) generates an adaptive immune response mediated by autoantibody that is produced by activated B cells and then induces atherosclerosis (Hermansson et al., 2010, Hilgendorf et al., 2014). However, in the present study, there was no correlation between lipid levels and atherosclerosis development. Therefore, we determined whether B2-cell activation is mediated by microbiota rather than by hyperlipidemia in atherosclerosis. Here, we confirmed, by eliminating the intestinal microbiota and depleting B2 cells in the WD-fed mice, that hyperlipidemia did not directly potentiate atherosclerosis by altering B2-cell activation. Our results provide novel evidence that B2 cells are causally related to microbiota-induced atherosclerosis. Our findings also indicate that proatherogenic effects do not solely depend on hypercholesterolemia-induced immune response. Clinically, these findings may explain why only control of lipids is not effective preventive therapy in some patients with atherosclerosis.

B cells play a complex role in the development of atherosclerosis via antibody production. In particular, the role of B2 cells in atherosclerosis is highly debatable (Kyaw et al., 2010, Tsiantoulas et al., 2014). As such, understanding the impact of B2 cells on atherosclerosis and elucidating factors that regulate their activity are important. In the present study, our observed differences in FO B cells of PVAT between the WD group and WD-AT group suggest that FO B cells are missing from the PVAT in the WD-AT group, potentially due to commensal microbe deletion by broad-spectrum antibiotics. However, it remains controversial whether commensal microbes directly induce B2-cell activation during the development of atherosclerosis and whether activated B2 cells play a critical role in this process. Thus, future studies are needed to determine whether B2 cells are essential for commensal microbe-derived atherogenesis and to elucidate potential atherogenic pathways. It is known that one of the early consequences of B2 cell activation is the up-regulation of costimulatory molecules, such as MHC class II molecules, which can serve to enhance B cell interactions and present antigen (Jiang et al., 2013, Jin et al., 2014). Another major outcome of B2-cell activation is the production of large amounts of specific antibodies (Shapiro-Shelef and Calame, 2005). The results of our study apparently showed that a greater proportion of B2 cells expressed MHC class II molecules in the spleen of WD-fed mice. We also detected elevated levels of IgG. In particular, the IgG subclass IgG3 displayed the highest activity in serum, whereas AT significantly reduced the expression of MHC class II molecules and the levels of IgG and IgG3. These results support the concept that microbiota-induced atherosclerosis is associated with B2-cell activation.

It is thought that B cells can be activated by stimuli such as danger-associated and pathogen-associated molecular patterns via TI processes. Activated B cells then modify responses such as antigen presentation and antibody production (Vinuesa and Chang, 2013). In general, as one of the pathogen-associated molecular patterns, commensal microbes or commensal microbe-derived LPS or peptidoglycan can be selectively recognized by the host's innate immune TLRs (Kawai and Akira, 2010). In fact, many studies have demonstrated that TLRs are contributors to atherosclerosis development (Edfeldt et al., 2002). As mentioned above, B cells are known to express TLRs (Nickerson et al., 2010, Pasare and Medzhitov, 2005). However, the functions of TLRs are traditionally linked to their effect on macrophages or dendritic cells, and the role of TLR signal pathway in B2 cells during the development of atherosclerosis remains to be fully elucidated. In our study, the up-down regulation of several TLR pathway-related genes that encode inflammation-related molecules in B2 cells suggests that B2-cell activation is driven by the microbiota via B2-cell TLR signaling pathway during atherosclerosis. Taken together, these results intriguingly suggest that under hyperlipidemic conditions, signals driven by the microbiota via TLR signaling pathway cause B2 cells to become functionally active and then generate active antibodies, cytokines, and chemokines, thereby contributing to the development of atherosclerosis.

Although B2 cells are not the only cell type that mediates atherogenesis, the results of the present study collectively demonstrate that B2 cells play an essential role in promoting commensal microbe-derived atherosclerosis, and consequently, the entire cascade leading to further acceleration of disease development in ApoE KO mice. We therefore suggest that future therapies could operate by shifting the homeostatic balance of B2 cells or their activation status. Actually, in the present study, although AT normalized the overly active B2 cell population in atherosclerosis, the extent to which this treatment affects the immune system or other immunocytes is open to future investigations. Therefore, in the present study, we then further tested definitively whether B2-directed therapies, such as ablation of B2 cells with a B2 cell-depleting, anti-mouse CD23 antibody, could serve as a therapeutic target in atherosclerosis. CD23 is the low-affinity IgE Fc receptor expressed on mature resting conventional B2 cells, but not on B1 cells or T cells. CD23 is a B2-cell-specific antigen, and it is now known that the functions of CD23 include a role in B2 cell growth, antigen presentation, germinal center B2 cell survival, prothymocyte maturation, myeloid precursor proliferation, and, possibly, a role in protecting cells from oxidative stress (Stief et al., 1994). Moreover, recent studies also have demonstrated that CD23 in B2 cells binding to macrophages leads to a pro-inflammatory pattern of activation and cytokine secretion (Dasic et al., 1999, Lecoanet-Henchoz et al., 1995). In the present study, anti-CD23 antibody-treated mice had markedly reduced numbers of B2 cells but an otherwise relatively intact immune system. Confirming this, we showed that under physiological (i.e., ND) conditions, anti-CD23 antibody treated-mice were virtually devoid of B2 cells in spleen. In contrast, the numbers of T cells and macrophages were largely unaffected in spleen. Therefore, these findings suggest that pharmacological depletion of B2 cells within the WD-fed mouse results in reduced susceptibility to atherosclerosis mediated by the commensal microbe-specific activation of B2 cells. Further studies of B2-cell ablation in normal human subjects and in patients are required to establish the translational value of our findings, which could potentially lead to important therapeutic approaches. In addition, as mentioned above, in future studies using our model it would be of interest to evaluate the effects of increasing the dietary intake of precursors of toxic metabolic products of the intestinal microbiome on the development of atherosclerosis. Such effects might act independently of, or perhaps be mediated by, the B2-cell effects on atherosclerosis that we have described.

In summary, our results provide evidence for a hitherto unrecognized pathway of immune activation in atherosclerosis, which driven by B2 cell that sense microbiota via TLRs pathway.

The following are the supplementary data related to this article.

Supplementary Table S1 RT^2^ Profiler PCR array: Gene list related to TLRs signaling (sheet 1); List of fold changes in the expression of genes relevant to TLRs signaling pathway in FO B cells (sheet 2); List of fold changes in the expression of genes relevant to TLRs signaling pathway in MZ B cells (sheet 3). Supplementary Table S1

Supplementary figures Image 2

## Funding Sources

T.I. is supported by a Grant-in-Aid for Scientific Research from the Ministry of Education, Culture, Sports, Science, and Technology (MEXT) no. 26461257, and Yokohama Foundation for Advancement of Medical Science. L.C. is supported by MEXT Government Scholarship no. 122229. These funding sources had no role in the study design; in the collection, analysis and interpretation of data; in the writing of the manuscript; or in the decision to submit the paper for publication.

## Conflicts of Interest

The authors declare no conflict of interest.

## Author Contributions

L.C. planned and performed experiments and wrote the manuscript. L.C. and R.S. performed molecular experiments. L.C. and T.K. performed in vivo experiments. L.C., T.K., and M.D. performed inhibitor experiments. S.M. provided reagent. T.I. helped design experiments, reviewed the manuscript, and conceived the scientific ideas and edited the manuscript. S.U., the former principal investigator, oversaw the project.

## Figures and Tables

**Table 1:** Relative fold changes in the expression of genes relevant to TLR signaling pathway in FO B cells.

Gene	Fold regulation (compared with spleen FO B cells of WD group)	
	FO B cells of spleen (WD-AT group)	FO B cells of PVAT (WD group)
*Ccl2*	− 1.1481	17.8381
*Cd14*	1.993	7.189
*Cebpb*	3.2331	1.1982
*Fos*	1.726	− 3.5108
*Hspa1a*	− 5.1166	9.3761
*Il1b*	− 1.0307	3.3433
*Il1r1*	1.2616	3.5637
*Jun*	1.2743	− 2.16
*Nfkbib*	2.8025	1.5068
*Ptgs2*	− 2.3499	3.9493
*Tnfrsf1a*	1.5827	2.7597

**Table 2:** Up-down regulation in the expression of genes relevant to TLRs signaling pathway in MZ B cells in the WD group versus WD + AT group.

Gene	Fold regulation in MZ B cells (compared with WD group)
*Chuk*	3.8971
*Fos*	2.6863
*Hspa1a*	− 2.3842
*Ikbkb*	2.6361
*Irf1*	2.0659
*Mapk8*	2.6917
*Mapk8ip3*	6.4522
*Mapk9*	3.1227
*Tlr5*	− 2.4133
*Tollip*	2.1339
*Tradd*	3.121
*Traf6*	3.3725
