# Supplementary material for: Commensal Microbe-specific Activation of B2 Cell Subsets Contributes to Atherosclerosis Development Independently of Lipid Metabolism
Source: eBioMedicine. 2016 Oct 20;13:237–47. doi: 10.1016/j.ebiom.2016.10.030 (PMC5264349; doi:10.1016/j.ebiom.2016.10.030)
Supplement: Supplementary file 2 — Supplementary figures [file mmc2.pdf]

Supplementary Information

Commensal microbes-specific activation of B2 cell subsets contribute to atherosclerosis development independent of lipid metabolism

Lin Chen[1], Tomoaki Ishigami[1*], Rie Nakashima-Sasaki[1], Tabito Kino[1], Hiroshi Doi[1], Shintaro Minegishi[1], Satoshi Umemura[1]

*Correspondence to: tommmish@hotmail.com.

Supplementary Figure legends

Supplementary Figure S1 | Characteristics of C57BL/6J wild type mice following WD and AT. (A) Body weights. AT: antibiotic treatment; WD: western diet; ND: normal chow diet. Data are depicted as mean ± SEM. Statistical significance was calculated by 1-way ANOVA. $^{**}p<0.001$. n=5 for each group. **(B)** Weight of spleen was showed as a percentage of body weight. Results are presented as mean± SEM. n=5 per group. **(C and D)** Micro-CT of total percent of visceral fat volume (C) and subcutaneous fat volume (D). Data are representative as means ± SEM. n=5 for each group. $^{*}p<0.05$, $^{**}p<0.001$ according to the post hoc ANOVA statistical analysis. **(E-H)** Serum levels of total cholesterol (E), LDL (F) and HDL cholesterol (G) and triglyceride (H) were assessed. LDL: low density lipoprotein; HDL: high density lipoprotein. Results are presented as mean ± SEM. n=5 per group, $^{*}p<0.01$, $^{**}p<0.001$.

Supplementary Figure S2 | Typical features of spleen in each group. (A) Weight of spleen was assessed at the end of the study and showed as a percentage of body weight. **(B and C)** Spleen sections were stained with HE (B) and numbers of germinal centers were also quantified (C). The scale bar indicates 300 μm. Data are depicted as mean ± SEM. Statistical significance was calculated by 1-way ANOVA. *$p<0.05$. n=7 for each group.

Supplementary Figure S3 | Effect of antibiotic treatment on B2 cell numbers in PVAT and spleen from C57BL/6J wild type mice. (A and B) Flow cytometry analysis of (A) follicular (FO) B cells of PVAT, (B) FO B cells and marginal zone (MZ) B cells in spleen, respectively (n=5 mice per group). Cell counts are presented as mean± SEM. *$p<0.05$, **$p<0.01$.

Supplementary Figure S4 | Effect of an anti-mouse CD23 antibody on T cell and macrophage numbers in spleen. (A and B) Total T cell and macrophage numbers in the spleen of mice treated with saline or mouse specific CD23 antibody. Representative flow cytometric plots on the left (A) and group data on the right (B). Values are presented as mean ± SEM from n=6 experiments.

Supplementary Tables

Supplementary Table S1. RT^2 Profiler PCR array: Gene list related to TLRs signaling (sheet 1); List of fold changes in the expression of genes relevant to TLRs signaling pathway in FO B cells (sheet 2); List of fold changes in the expression of genes relevant to TLRs signaling pathway in MZ B cells (sheet 3).

Figure S1

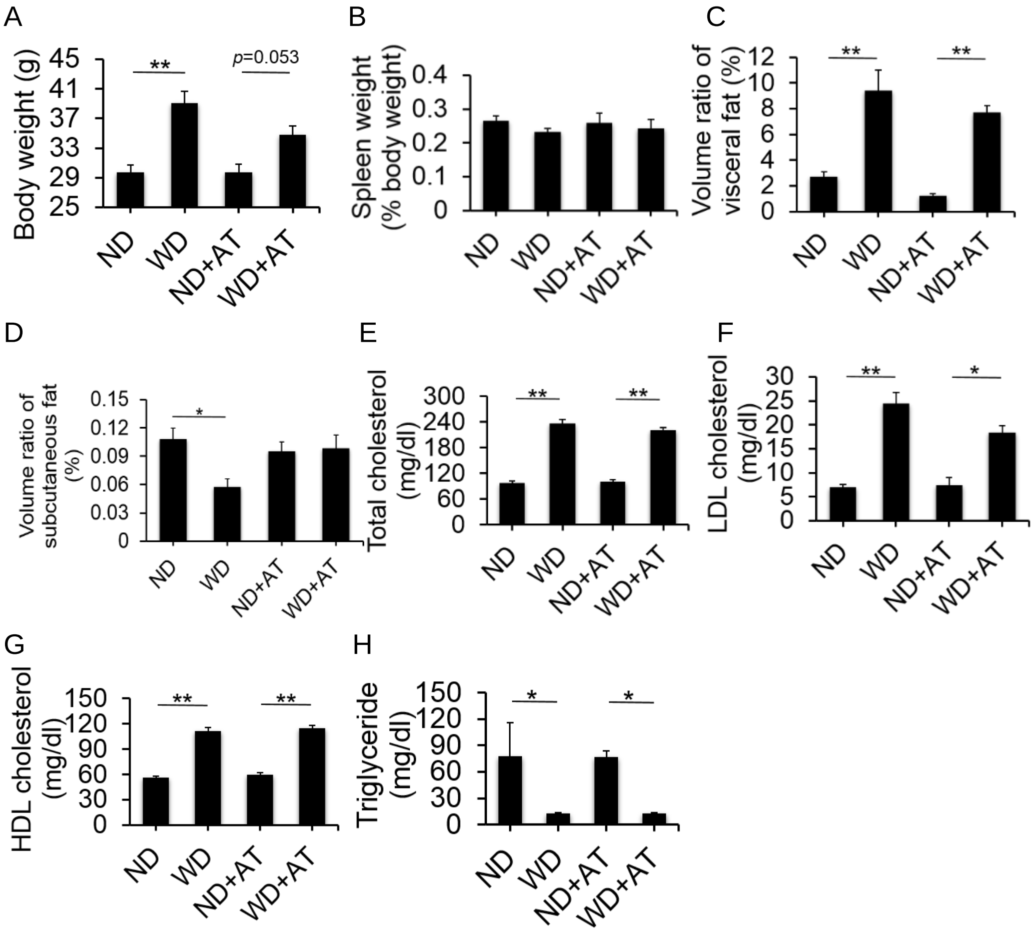
A
B
C
D
E
F
G
H
Body weight (g)
Spleen weight (% body weight)
Volume ratio of visceral fat (%)
Volume ratio of subcutaneous fat (%)
Total cholesterol (mg/dl)
LDL cholesterol (mg/dl)
HDL cholesterol (mg/dl)
Triglyceride (mg/dl)
p=0.053
ND
WD
ND+AT
WD+AT

Figure S2

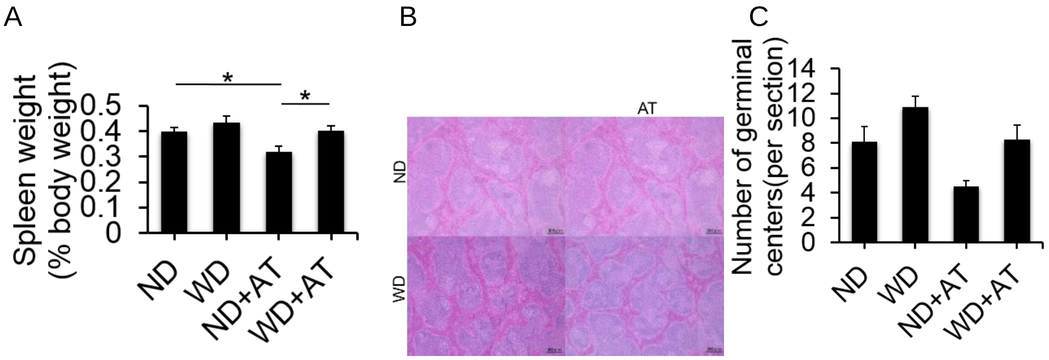
A
Spleen weight
(% body weight)
0.5
0.4
0.3
0.2
0.1
0
*
*
ND
WD
ND+AT
WD+AT
B
AT
ND
WD
C
Number of germinal
centers(per section)
14
12
10
8
6
4
2
0
ND
WD
ND+AT
WD+AT

Figure S3

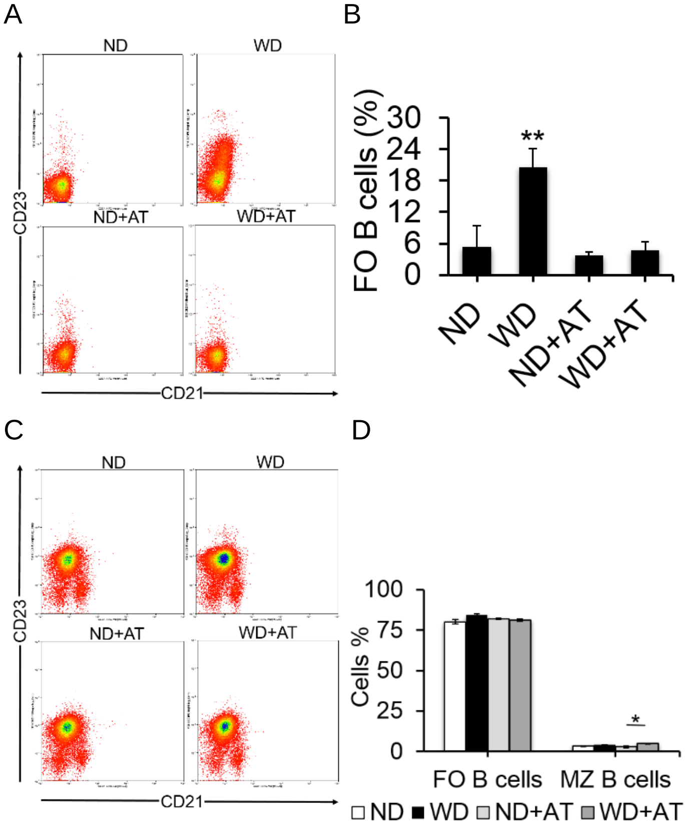
A
ND
WD
ND+AT
WD+AT
CD23
CD21
B
FO B cells (%)
30
24
18
12
6
0
**
ND
WD
ND+AT
WD+AT
C
ND
WD
ND+AT
WD+AT
CD23
CD21
D
Cells %
100
75
50
25
0
*
FO B cells
MZ B cells
ND
WD
ND+AT
WD+AT

Figure S4

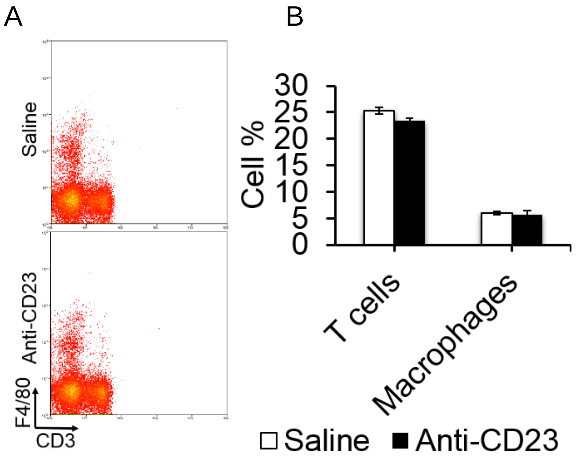
A
Saline
Anti-CD23
F4/80
CD3
B
Cell %
30
25
20
15
10
5
0
T cells
Macrophages
Saline
Anti-CD23
